# 18F-fluorodeoxyglucose (FDG) PET/CT after two cycles of neoadjuvant therapy may predict response in HER2-negative, but not in HER2-positive breast cancer

**DOI:** 10.18632/oncotarget.5001

**Published:** 2015-08-21

**Authors:** Jingyi Cheng, Yujie Wang, Miao Mo, Xiao Bao, Yingjian Zhang, Guangyu Liu, Jun Zhang, Daoying Geng

**Affiliations:** ^1^ Department of Radiology, Huashan Hospital, Fudan University, Shanghai, P.R. China; ^2^ Department of Breast Surgery, Key Laboratory of Breast Cancer in Shanghai, Fudan University Shanghai Cancer Center, Department of Oncology, Shanghai Medical College, Fudan University, Shanghai, P.R. China; ^3^ Clinical Statistics Center, Fudan University Shanghai Cancer Center, Department of Oncology, Shanghai Medical College, Fudan University, Shanghai, P.R. China; ^4^ Department of Nuclear Medicine, Fudan University Shanghai Cancer Center, Department of Oncology, Shanghai Medical College, Fudan University Shanghai, P.R. China

**Keywords:** breast cancer, neoadjuvant therapy, trastuzumab, FDG PET/CT

## Abstract

The aim of this prospective study was to assess the ability of 18F-fluorodeoxyglucose (^18^FDG) positron emission tomography/computed tomography (PET/CT) scanning to predict pathological complete response (pCR) in breast cancer, and to investigate whether timing of the scan and trastuzumab treatment influence the accuracy of pCR prediction in human epidermal growth factor receptor 2 (HER2) positive breast cancer patients. We treated 81 locally advanced breast cancer patients with four cycles of neoadjuvant chemotherapy (NAC). HER2-negative breast cancer patients received NAC alone, while HER2-positive breast cancer patients received NAC plus trastuzumab. ^18^FDG PET/CT scans were scheduled at baseline and after the second cycle of NAC. Axillary lymph node (ALN) dissection was performed after the last cycle of neoadjuvant therapy. Relative changes in standardized uptake values (SUV) between the two PET/CT scans (ΔSUV) in primary tumors and ALN metastases were calculated. There were 75 patients with 150 PET/CT scans in the final analysis, including 41 HER2-negative and 34 HER2-positive cases. In the HER2-negative group, the ΔSUV predicted overall and ALN pCR; the receiver operating characteristics-areas under curve (ROC-AUC) were 0.87 and 0.80 (*P* = 0.0014 and 0.031, respectively) and the negative predictive values were 94% and 89% respectively. However, in the HER2-positive group, ΔSUV could predict neither overall nor ALN pCR; the ROC-AUCs were only 0.56 and 0.53, with *P* = 0.53 and 0.84, respectively. Hence, the ΔSUV after two cycles of neoadjuvant therapy could predict pCR in HER2-negative patients treated with NAC alone, but not in HER2-positive patients treated with NAC plus trastuzumab.

## INTRODUCTION

Preoperative neoadjuvant therapy is used as a standard treatment for locally advanced breast cancer to downstage the primary tumor and increase the breast conservation rate. Early prediction of success of neoadjuvant therapy is critical for determining whether a current treatment should be continued, stopped, or changed to a more aggressive regimen [1–3]. In recent years, several studies demonstrated that 18F-fluorodeoxyglucose (^18^FDG) positron emission tomography/computed tomography (PET/CT) is a useful tool for evaluating NAC response [4–6]. Relative change in the standardized uptake value (ΔSUV) after one or two cycles of NAC was reported to be a strong indicator of pathological response [7]. However, treatment options and responses to therapy differ greatly among various phenotypes. These differences complicate the utilization of ΔSUV as a predictor of pathological responses [8–12].

This prospective study investigated the predictive reliability of ^18^FDG uptake change in patients with human epidermal growth factor receptor 2 (HER2)-negative and HER2-positive phenotypes, particularly with respect to axillary lymph node (ALN) metastasis.

## RESULTS

### Patients and pathologic response

Eighty-one breast cancer patients were recruited: 3 were excluded because they missed follow-up (1 triple-negative breast cancer (TNBC) and 2 HER2-positive) and 3 HER2-positive patients underwent NAC only (including 1 luminal with HER[3+]) and 2 HER2-positive). Ultimately, 75 patients were included in the final analysis. Breast cancer was confirmed through core needle biopsy. Patients’ tumor characteristics and treatments are listed in Table 1.

**Table 1:** Patient and Tumor Characteristics (*n* = 75)

**Age (years)**		
Mean	43.7	
SD	12.35	
Range	24–65	
**Tumor histology**		
Ductal	72	96.0%
Lobular	3	4.0%
**Clinical tumor classification**		
T1	10	13.3%
T2	43	57.3%
T3	19	25.3%
T4	3	4.0%
**Clinical lymph node classification**		
N0	22	29.3%
N1	39	52.0%
N2	14	18.6%
**primary lesions (*n* = 75)**		
pCR	33	44.0%
non-pCR	42	56.0%
**axillary lymph node metastasis (*n* = 53)**		
pCR	30	56.6%
non-pCR	23	43.4%
**Overall (primary + axillary lymph node metastasis) (*n* = 75)**		
pCR	27	36.0%
non-pCR	48	64.0%
HER2-positive group (*n* = 34)	pCR	non-pCR
ER/PR(−) HER2(+) (*n* = 23)	12	11
ER/PR(+) HER2(+) (*n* = 11)	7	4
HER2-negative group (*n* = 41)	pCR	non-pCR
ER/PR(−) HER2(−) (*n* = 13)	4	9
ER/PR(+) HER2(−) (*n* = 28)	4	24

Abbreviations: SD, standard deviation; ER, estrogen receptor; PR, progesterone receptor. HER2, human epidermal growth factor receptor 2; NAC neoadjuvant chemotherapy; pCR, pathologic complete response.

A majority of tumors were luminal (52.0%; 39/75), followed by HER2-positive (30.6%; 23/75), and TNBC (17.3%; 13/75). pCR occurred more often in patients with HER2 positive tumors (52.1%) than in those with TNBC and luminal tumors (30.7% and 28.2%, respectively). Furthermore, the HER2-positive group treated with NAC plus trastuzumab showed higher pCR rates than the HER2-negative group treated with NAC alone (55.8% and 19.5%, respectively).

### ΔSUVpeak in primary lesions and metastatic lymph nodes

A total of 150 PET/CT scans from 75 patients were eligible for analysis. Among them, 53 had ALN metastasis confirmed by both core needle biopsy and ^18^FDG PET/CT. In the baseline PET/CT scan, the SUV peak of the primary lesions ranged from 22.2 to 1.60 (mean = 8.14 ± 4.54). The SUVpeak of ALN metastasis ranged from 15.87 to 1.20 (mean = 6.35 ± 4.02). On follow-up PET/CT scans after two cycles of neoadjuvant therapy, the SUVpeak of the primary lesions ranged from 9.60 to 0 (mean = 2.88 ± 2.92), and that of ALN metastases ranged from 7.79 to 0 (mean = 1.67 ± 1.76).

In the HER2-positive group, the SUVpeak of primary lesions fell from a baseline of 7.95 ± 4.65 to 2.06 ± 3.01 in the subsequent scan, a decrease of 73% ± 32%. Moreover, the SUVpeak of ALN metastasis fell from a baseline of 6.46 ± 3.75 to 1.19 ± 1.70, a decrease of 84% ± 18%.

In the HER2-negative group, the SUVpeak of the primary lesions fell from a baseline of 8.28 ± 4.49 to 3.55 ± 2.63, a decrease of 52% ± 33%. The SUVpeak of ALN metastasis fell from 6.23 ± 4.30 to 2.20 ± 1.70, a decrease of 60% ± 31%. The *P* values were 0.005 and 0.001, respectively.

### ΔSUVpeak of primary lesions predict overall pCR

In all 75 patients, ^18^FDG PET/CT was performed before and after two cycles of neoadjuvant therapy. The SUVpeak obtained at these times could not predict overall pCR for primary lesions or ALN metastases. The ΔSUVpeak of the primary lesion had a moderate predictive value with a receiver operator characteristics-area under the curve (ROC-AUC) of 0.75 (95% confidence interval [CI] 0.65–0.86, *P* = 0.001); the sensitivity and specificity were 78% and 69%, respectively.

**Figure 1:**
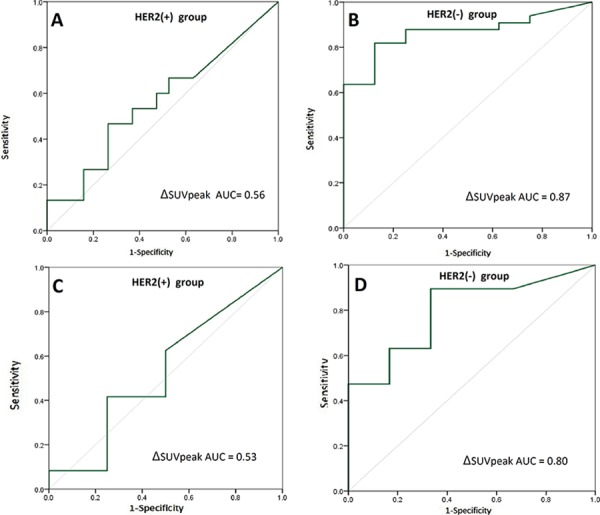
Receiver operating characteristics (ROC) analysis of ΔSUVpeak for the prediction of pathologic complete response (pCR) ROC analysis for prediction of overall pCR with the primary lesions’ ΔSUVpeak in HER2-positive breast cancer **A.** and HER2-negative breast cancer **B.** ROC analysis for prediction of axillary lymph node (ALN) pCR with ΔSUVpeaks of ALN metastases in HER2-positive breast cancer **C.** and HER2-negative breast cancer **D.** ΔSUVpeak refers to the difference in standard uptake values between baseline and after two chemotherapy cycles.

Of all cases, 34 were in the HER2-positive group that was treated with NAC and trastuzumab. ROC analysis showed that the ΔSUVpeak could not effectively predict the overall pCR because the ROC-AUC was only 0.56 (95% CI 0.36–0.76, *P* = 0.53). However, for the 41 patients of the HER2-negative group treated with NAC alone, ROC analysis showed that the ΔSUVpeak accurately predicted the overall pCR because the ROC-AUC was 0.87 (95% CI 0.75–0.98, *P* = 0.0014). The sensitivity, specificity, positive predictive value, negative predictive value, and accuracy were 75%, 85%, 60%, 94%, and 83%, respectively (Figure 1A, 1B).

**Figure 2:**
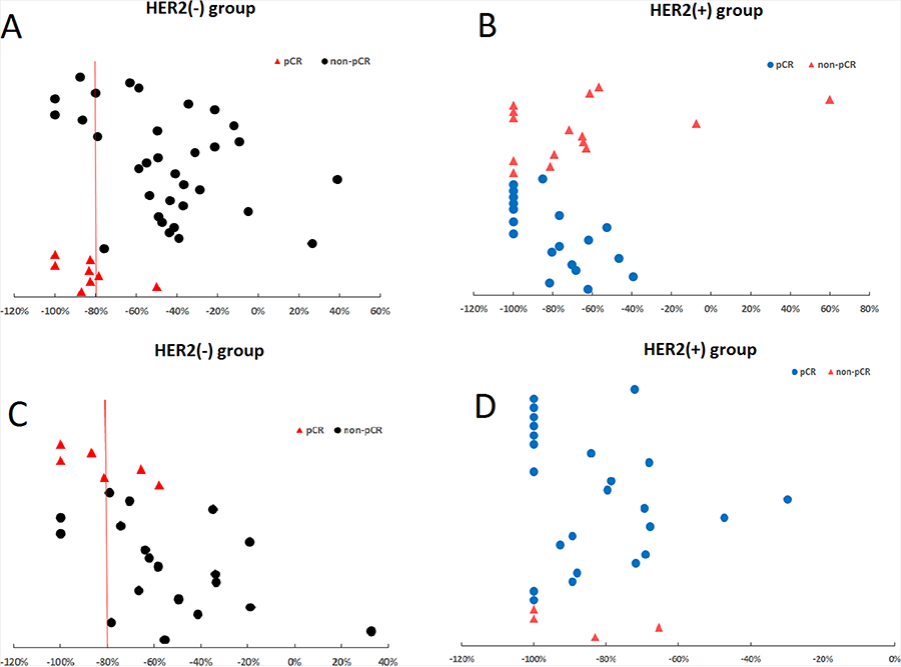
Pathological complete response (pCR) assessment in relation to the ΔSUVpeak Application of a cutoff ΔSUVpeak of >80% predicted 88% of overall non-pCRs **A.** and 89% of axillary lymph node (ALN) non-pCRs **C.** in the HER2-negative group, displaying higher negative predictive values of 94% and 89%, respectively. In the HER2-positive group, ΔSUVpeak could not predict overall **B.** or ALN pCRs/non-pCRs **D.** because a clear cutoff could not be defined. ΔSUVpeak refers to the difference in standard uptake values between baseline and after two chemotherapy cycles.

In the HER2-negative group, we predicted 75% of pCRs (6/8) and 88% of non-pCRs (29/33) by applying a cutoff value of ΔSUVpeak > 80%. However, in the HER2-positive group, the ΔSUVpeak could not predict pCR accurately because a clear cutoff could not be defined (Figure 2A, 2B).

### ΔSUVpeak of ALN metastasis predicts pCR

There were 53 patients with ALN metastasis confirmed by both core needle biopsy and ^18^FDG PET/CT. Neither the baseline nor post two-cycle SUVpeak alone could predict ALN pCR. The ΔSUVpeak of the ALN metastases had a moderate predictive value with an ROC-AUC of 0.74 (95% CI 0.60–0.88, *P* = 0.0021). The sensitivity and specificity were 87% and 61%, respectively.

Of all 75 cases, 28 were HER2-positive and were treated with NAC and trastuzumab. ROC analysis showed that the ΔSUVpeak of ALN metastasis could not predict the ALN pCR effectively because the ROC-AUC was only 0.53 (95% CI 0.20–0.86, *P* = 0.84). However, in the HER2-negative group of 25 cases treated with NAC alone, the ΔSUVpeak accurately predicted the ALN pCR, as the ROC-AUC was 0.80 (95% CI 0.60–0.99, *P* = 0.031). The sensitivity, specificity, positive predictive value, negative predictive value, and accuracy were 67%, 89%, 67%, 89%, and 84%, respectively (Figure 1C, 1D).

In the HER2-negative group, we could predict 66% of pCRs (4/6) and 89% of non-pCRs (17/19) by applying a cutoff value of ΔSUVpeak >80%. However, in the HER2-positive group, the ΔSUVpeak could not accurately predict pCR because a clear cutoff value could not be defined (Figure 2C, 2D).

## DISCUSSION

The use of ^18^FDG PET/CT imaging as an early predictive tool for breast cancer pCR has recently been encouraged. However, there has been controversy regarding the optimal timing of ^18^FDG PET/CT scanning. Most researchers advocate performing ^18^FDG PET/CT scanning halfway through chemotherapy [13, 14], while others suggest it be performed after the first cycle of chemotherapy [15, 16].

HER2-positive breast cancers usually undergo notable SUV changes during therapy, compared to other phenotypes. A 60% change in SUV is easily achievable after the first cycle of chemotherapy. This degree of change ought to be sufficient for pCR prediction; therefore, some researchers suggest conducting ^18^FDG after the first cycle of chemotherapy to determine whether switching the therapeutic regimen is warranted [17].

We showed that ΔSUV after two cycles of neoadjuvan therapy accurately predicted the response in HER2-negative, but not HER2-positive patients. This appears to contradict existing studies. Actually, our result reveals the same view point on scanning timing for HER2-positive from another aspect. The average degree of SUV change in the HER2-positive group was 73% ± 32%, which was much greater than the 52% ± 33% observed in the HER2-negative group (*P* = 0.005 for primary lesions and *P* = 0.001 for ALN metastases). This suggests that the optimal ^18^FDG PET-CT timing for the HER2-positive group, for purposes of predicting pCR, was missed.

**Figure 3:**
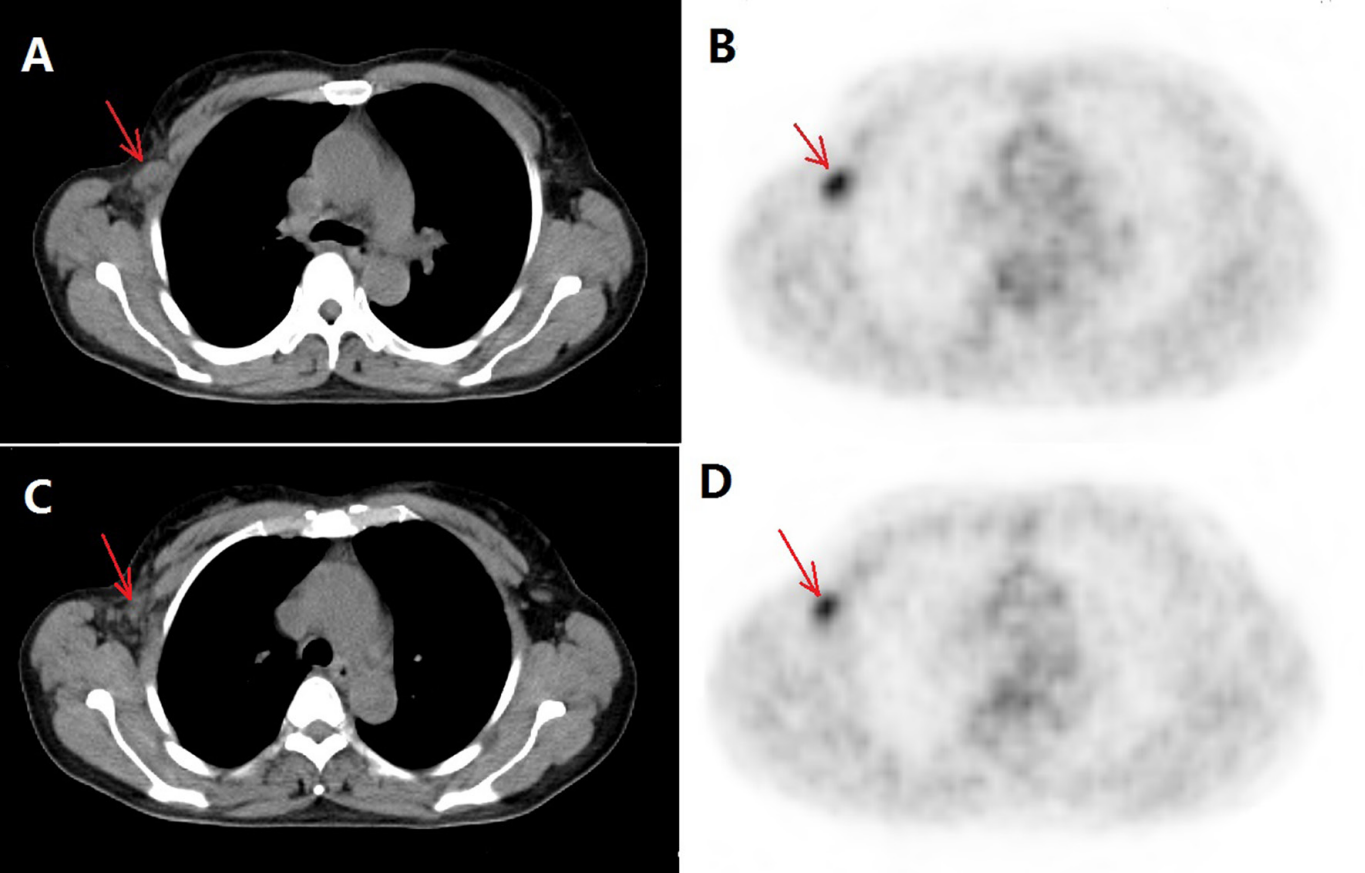
A false negative case involving a T2N1 breast primary lesion (HER2-positive) and right axillary lymph node metastasis before and after neoadjuvant chemotherapy (NAC) plus trastuzumab Transverse slices of CT and PET images show that the size of the right axillary lymph node (ALN) metastasis is 2.3 × 1.3 cm **A.** with high FDG uptake and a baseline SUVpeak of 5.16 **B.** After NAC plus trastuzumab treatment, the right ALN shrank **C.**; FDG uptake decreased and the SUVpeak was 2.72 **D.** The ΔSUVpeak is − 47% (from 5.16 to 2.72), less than the threshold of − 80%, but the result of ALN dissection was pathological complete response. ΔSUVpeak refers to the difference in standard uptake values between baseline and after two NAC cycles.

Another reason is that the inflammation reaction caused by trastuzumab influences predictive accuracy. Firstly, in addition to directly inhibiting intracellular signaling in HER2-positive tumor cells, the combination with trastuzumab induces antibody-dependent cellular cytotoxicity (ADCC), which also contributes to changes in SUV [14]. Secondly, the decrease in ^18^FDG uptake in patients receiving trastuzumab is not a pure reflection of cell killing but also reflects other specific effects on glucose metabolism, such as reductions in GLUT1 and hexokinase II activity [18, 19]. As a result, breast lesion and ALN metastasis shrink and FDG uptake decrease greatly after two cycles of NAC due to higher sensitivity for NAC in HER2-positive group. At the same time, inflammation reaction caused by trastuzumab appears, causing the SUVs of residual lesions to increase and ΔSUVs to therefore decrease. In cases with lower SUV baselines in particular, slight increases in SUV caused by inflammation could easily generate false negatives (Figure 3A–3D).

The response of breast cancer ALN metastases after NAC is of greater concern than that of the primary foci. In previous studies of HER2-positive cancers, the complete response rates of both primary foci and lymph nodes were quite high; hence, overtreatment can be avoided if ALN metastases show early pCR. Roussean et al. reported that the sensitivity and accuracy of therapeutic effect prediction for axillary lymph node metastases after chemotherapy were 96% and 84%, respectively, with an SUV decrease of 50% set as the threshold [11]. Similarly, Koolen et al. reported that the specificity and positive predictive values were 95% and 86%, respectively, if the SUV decrease was greater than 60% and the ROC-AUC was 0.80 [10]. There were also contradictory reports; Jung et al. reported that there was no correlation between SUV decrease and the pCR rate of lymph node metastasis [20]. In these reports, the breast cancer phenotypes were not considered, nor were the influences of therapies.

In our study, ΔSUV was shown to be a more valuable predictor for HER2-negative patients who underwent NAC alone (ROC-AUC = 0.80, *P* = 0.031), while the ROC-AUC of the ΔSUV decreased to 0.53 (*P* = 0.84) in HER2-positive patients who received NAC plus trastuzumab. Determining a cutoff value of ΔSUVpeak > 80% can predict 66% of pCR and 89% of non-pCR in HER2-negative patients. However, ΔSUVpeak did not accurately predict pCR in HER2-positive patients because a clear cutoff could not be established.

The main limitation of our study is the absence of SUV values after the first cycle of NAC; having multiple time points can better determine the best ^18^FDG PET-CT timing for assessment.

In conclusion, this study shows that response monitoring of breast cancer patients with ^18^FDG PET-CT during neoadjuvan therapy is dependent on both phenotypes, therapeutic regimens and scan timing. For HER2- negative patients treated with NAC alone, the optimal timing is after tow cycles. ΔSUV can accurately predict pCR. Application of a cutoff ΔSUVpeak of >80% predicted 88% of overall non-pCRs and 89% of ALN non-pCRs, displaying higher negative predictive values of 94% and 89%, respectively. The clinical benefit of this study is that ^18^FDG PET-CT could offer the opportunity to switch to a different regimen for an individual patient who is likely to be non-pCR to NAC.

## MATERIALS AND METHODS

### Patients

To assess the potential value of ^18^FDG PET/CT in predicting the response during neoadjuvant therapy, 81 breast cancer patients were recruited for this prospective study starting in February 2013. This study was approved by the Institutional Ethics Committee, and written informed consent was obtained from all patients. Enrolled were patients with newly diagnosed, non-inflammatory, and locally advanced (stage II and III) breast cancer who accepted neoadjuvant therapy. Clinical stage was determined according to the American Joint Committee on Cancer 6th edition. Tumor size and T stage were assessed by clinical examination, ultrasonography, and/or magnetic resonance imaging (MRI). Diagnoses of invasive breast carcinoma and ALN metastasis were confirmed by core needle biopsy and fine needle aspiration, respectively, in all patients. Clinical stages were determined by mammography, ultrasonography, and MRI according to the TNM (tumor-node-metastasis) classification. Exclusion criteria were pregnancy, breast surgery, chemotherapy or radiotherapy history, diabetes, age younger than 18 years old, no ^18^FDG uptake at baseline, or ineligibility for surgery.

### ^18^FDG PET/CT Protocol

^18^FDG was produced by the Cyclotron (Siemens CTI RDS Eclipse ST) and Explora FDG4 modules at our center. All patients were required to fast for at least 6 hours to ensure glucose blood levels below 10 mmol/L (180 mg/dL), which was a prerequisite for undergoing the procedure. As long as the patient's blood glucose level was over 10 mmol/L(180 mg/dL), the PET/CT exam should be cancelled. Before and after injection, patients lay comfortably in a quiet, dimly lit room. Scanning was initiated 1 hour after administration of the tracer (7.4 MBq/kg or 0.2 mCi/kg). The data acquisition procedure was as follows: CT scanning was first performed from the proximal thighs to head with 120 kV, CARE dose 4D mode, 80–250 mA, pitch 3.6. Intravenous or oral contrast was not used in CT scans. Immediately after CT scanning, a PET emission scan that covered the identical transverse field of view was obtained. Acquisition time was 2–3 min per table position. PET image data sets were reconstructed iteratively by applying the CT data for attenuation correction, and co-registered images were displayed on a workstation.

The baseline ^18^FDG PET/CT scans were scheduled prior to initiation of neoadjuvant therapy, at least ten days after core biopsy. The second scans were scheduled after the completion of the second cycle of neoadjuvant therapy just before commencing the third cycle. The same acquisition parameters were used for baseline and halfway studies. All the patients’ PET examinations were done at the same center with the same equipment and methods. The PET/CT data was interpreted by two nuclear physicians blinded to clinical, radiologic, and pathologic findings.

### Image analysis

Several lesions were evaluated, including primary lesions within the breast and regional lymph node metastases. All images were analyzed on a clinical Leonardo workstation with TrueD software. All the lesion volumes were defined by manually defining the volume of interest (VOI), referred to as the FDG-VOI on the^18^FDG PET/CT, and the hottest spot in the tumor foci was noted as the peak values of SUV (SUVpeak). After evidence of the initial ^18^FDG hyper-metabolism subsided on the PET/CT acquisition during neoadjuvant therapy, the VOI was copied onto the images of the second scans. The lesion with the highest initial uptake was assessed. The ALN to evaluate was defined as that having the most intense activity (SUVpeak ≥ 2.5) in the axilla [21]. Relative changes in SUVpeak between two PET/CTs (ΔSUVpeak) were calculated according to the following formula:

ΔSUVpeak = [(SUVpeak of second PET/CT − SUVpeak of baseline PET/CT) / SUVpeak of baseline PET/CT] × 100%.

### Histopathological analysis, definition of breast cancer subtypes and treatment

Breast cancer diagnosis was performed via core-needle biopsy. Tumor grade was determined using the modified Scarff-Bloom-Richardson classification. Estrogen receptor (ER), progesterone receptor (PR), and HER2 status was determined on representative paraffin sections from each tumor using immunohistochemical (IHC) staining. ER and PR expression was considered positive if the number of positive nuclei was > 1%. Cytoplasmic staining was not evaluated for consistency with the literature [22]. Positivity for the HER2 protein was evaluated according to the criteria of the HercepTest [22, 23]. The HER2 membrane staining intensity and pattern were evaluated using the 0 to 3+ scale. Scores of 0 and 1+ (weak immunostaining in less than 10% of tumor cells) were defined as negative (HER2[−]), 2+ (complete membrane staining in at least 10%, but less than 30%, of tumor cells) as equivocal, and 3+ (uniformly intense membrane staining in at least 30% of tumor cells) as positive (HER2[+]). Tumors with IHC scores of 3+ and tumors with 2+ that were further tested by fluorescence *in situ* hybridization (FISH) were reclassified as HER2(+). Tumors with IHC scores of 0 or 1+ and tumors with IHC scores of 2+ that were FISH-negative were classified as HER2(−).

Using IHC, the tumors were further classified into three subtypes:

- Luminal: ER and/or PR positive, HER2(−) or HER2(+) (*n* = 39).
- HER2(+): ER and PR negative, HER2(+), (*n* = 23).
- TNBC: ER, PR, and HER2(−) (*n* = 13).

Different therapeutic regimens were administered according to subtypes and HER2 status:

- HER2(+) group: Luminal with HER2(3+) or HER2(2+) that were FISH-positive and HER2-positive (*n* = 34). For HER2-positive patients, four weekly cycles of paclitaxel 80 mg/m^2^ and carboplatin at AUC of 2 in combination with trastuzumab 2 mg/kg (on days 1, 8, 15, and 22; loading dose 4 mg/kg for week 1) were administered, and ALN dissection was performed less than 4 weeks after the last cycle of chemotherapy.
- HER2(−) group: Luminal with HER2(0), HER2(1+), or HER(2+) that were FISH negative and TNBC (*n* = 41). Patients with HER2-negative breast cancer received four weekly cycles (12 doses over 16 weeks) of paclitaxel 80 mg/m^2^ and carboplatin at AUC of 2 (both on days 1, 8, and 15 of a 28-day cycle) followed by ALN dissection surgery.

### Pathology assessment

pCR was defined as the absence of residual invasive cancer on hematoxylin and eosin evaluation of the complete resected breast specimen and all sampled regional lymph nodes following completion of neoadjuvant systemic therapy. Absence of *in situ* carcinoma was not required to define pCR [24].

### Statistical analysis

Data was analyzed using the SPSS 22.0 software. Correlation between texture parameters was analyzed using Person's test. The predictive performance regarding the identification of responders or non-responders was evaluated by using univariate ROC analysis. The ROC-AUCs were compared using the *Z* test. *P* < 0.05 was considered statistically significant.
